# A Phase II Trial of Bevacizumab in Patients with Recurrent/Progressive Solid Tumor Brain Metastases That Have Progressed Following Whole-Brain Radiation Therapy

**DOI:** 10.3390/cancers16112133

**Published:** 2024-06-04

**Authors:** Karan Dixit, Lauren Singer, Sean Aaron Grimm, Rimas V. Lukas, Margaret A. Schwartz, Alfred Rademaker, Hui Zhang, Masha Kocherginsky, Sofia Chernet, Laura Sharp, Valerie Nelson, Jeffrey J. Raizer, Priya Kumthekar

**Affiliations:** 1 Department of Neurology, Northwestern University, Chicago, IL 60611, USA; karan.dixit@nm.org (K.D.); rimas.lukas@nm.org (R.V.L.); mwalsh@nm.org (M.A.S.); sofia.chernet@nm.org (S.C.); jeffrey.raizer@nm.org (J.J.R.); 2 Department of Neurology, University of Chicago, Chicago, IL 60611, USA; lauren.singer@bsd.uchicago.edu; 3 Department of Neuro-Oncology, Rush University, Chicago, IL 60611, USA; sean_grimm@rush.edu; 4 Division of Biostatistics, Department of Preventive Medicine, Northwestern University, Chicago, IL 60611, USA; rademaker@northwestern.edu (A.R.); hzhang@northwestern.edu (H.Z.); mkocherg@northwestern.edu (M.K.); laura.sharp@northwestern.edu (L.S.); 5 Department of Hematology and Oncology, Northwestern University, Chicago, IL 60611, USA; valerie.nelson@nm.org

**Keywords:** bevacizumab, brain metastasis, whole-brain radiotherapy, survival

## Abstract

**Simple Summary:**

For patients with solid tumor brain metastases that progress after whole-brain radiotherapy, there are limited treatment options. The aim of our prospective trial was to examine the usage of bevacizumab as salvage therapy in this specific patient population, with primary endpoints being radiologic response, survival, safety, and quality of life. Our data show that bevacizumab was well tolerated, maintained quality of life, and improved overall survival with radiologic response.

**Abstract:**

Patients with solid tumor brain metastases that progress after whole-brain radiation have limited options. This prospective trial investigated the efficacy, safety, and tolerability of bevacizumab as salvage therapy in this population. Eligible patients received bevacizumab 10 mg/kg intravenously every 2 weeks until progression. The primary endpoint was radiologic response using Response Assessment in Neuro-Oncology (RANO) criteria. The secondary endpoints were progression-free survival (PFS), overall survival (OS), duration of response, and safety. Quality of life (QOL) was studied using the Functional Assessment of Cancer Therapy-Brain (FACT-Br) scale. Twenty-seven patients were enrolled, with twenty-four having evaluable data for response. The majority of histologies (n = 21, 78%) were breast cancer. The remaining histologies were non-small-cell lung cancer (n = 4, 15%), neuroendocrine cancer (n = 1, 3%), and papillary fallopian serous adenocarcinoma (n = 1, 3%). Eighteen patients had radiologic response, with two patients demonstrating partial response (8.33%) and sixteen patients demonstrating stable disease (66.7%). The median duration of response was 203 days. PFS at 6 months was 46%, median PFS was 5.3 m, and median OS was 9.5 m. Treatment was well tolerated, with six patients experiencing grade 3 lymphopenia and hypertension. There was one grade 3 thromboembolism. QOL was not negatively impacted. Bevacizumab is a safe and feasible salvage treatment with durable response and favorable overall survival for patients with progressive brain metastases after whole-brain radiation.

## 1. Introduction

Brain metastases (BMs) are the most common intracranial tumors, occurring in up to 20–30% of patients with solid malignancies, with an increasing incidence due to improving systemic therapy and prognosis, as well as greater use of surveillance imaging [1]. The most common solid tumor cancers that metastasize to the brain are lung, breast, melanoma, and renal cell [1]. In general, prognosis for patients with brain metastases is poor, with survival ranging from 3 to 11 months with treatment [2]. Patients with brain metastases are also more likely to have neurologic deficits which can have a significant impact on functional status and quality of life. Management depends on several factors including the number of lesions, tumor type, systemic disease burden, neurologic symptoms at presentation, and resectability, amongst others. The mainstays of treatment options have been surgical resection, stereotactic radiosurgery, and whole-brain radiotherapy (WBRT) [3]. Most systemic agents have limited blood–brain barrier penetration; however, immunotherapy and small-molecule genomically targeted agents are becoming increasingly important [4].

Patients with brain metastases often present with multiple lesions at diagnosis, limiting the extent of focal therapies such as resection and stereotactic radiosurgery. In patients with multiple brain metastases, WBRT has been the standard of care treatment since the 1950s. Although WBRT is effective for palliation, intracranial disease progression is common, and prognosis is limited, with a median survival of 3–7 months [2]. Treatment options for patients who progress after WBRT are limited. Stereotactic radiosurgery is an option in patients with favorable performance status, well-controlled systemic disease, and a limited volume of brain metastases [5]. Multiple cytotoxic systemic agents have been studied including high-dose methotrexate, temozolomide, topotecan, and lapatinib with capecitabine, with modest benefits [6]. There are limited data on the use of small-molecule targeted agents and immunotherapy specifically in the post-WBRT setting; however, given their response in brain metastases, they are an attractive option but are limited to a small number of molecular subgroups [7,8,9,10].

Bevacizumab (Bev), a human vascular endothelial growth factor (VEGF) inhibitor, has been studied and is approved for multiple tumor types, including non-small-cell lung cancer, colorectal cancer, renal cancer, genitourinary cancers, and recurrent glioblastoma. Prior studies with Bev in patients with brain tumors have not demonstrated any significant increased risk of toxicities [11]. Furthermore, patients with brain metastases often suffer from symptomatic peritumoral vasogenic edema, requiring corticosteroids which have numerous side effects and can be deleterious to patient quality of life and even survival, especially in patients on immunotherapy, as steroids can have an immunosuppressive effect [12]. Bev has demonstrated significant benefit in peritumoral edema control, including patients with brain metastases, often allowing for the weaning off of corticosteroids [13]. Thus, we performed a phase 2 study assessing the radiologic response, clinical response, as well as safety and tolerability of Bev as salvage therapy in patients with progressive or recurrent brain metastases after whole-brain radiotherapy. 

## 2. Materials and Methods

### 2.1. Patient Eligibility

Adult patients (age > 18) with histologically proven non-central nervous system primary solid malignancy at the time of diagnosis were eligible. Patients were enrolled from November 2013 to January 2017. Unequivocal radiological evidence of progression/recurrence on contrast-enhanced magnetic resonance imaging (MRI) after treatment with WBRT was required, with at least one lesion measuring ≥5 mm unidimensionally. Patients must have completed WBRT at least 12 weeks prior to enrollment to limit the risk of pseudoprogression. However, the development of a new lesion after 4 weeks from WBRT also qualified as disease progression. Patients must have been >4 weeks from any major surgery given the impacts of Bev on wound healing. Patients who underwent stereotactic radiosurgery to treat a progressive lesion after WBRT required unequivocal evidence of progression on advanced imaging with either MRI spectroscopy, MRI perfusion, positron emission tomography (PET), or tissue confirmation to be eligible. Patients could continue systemic therapy if they had isolated central nervous system (CNS) progression. There was no limit on the prior number of CNS-directed therapies. Other requirements included a Karnofsky Performance Status (KPS) of ≥60, a life expectancy of ≥12 weeks, and adequate hematologic levels (hemoglobin ≥ 10 gm/dL, platelets ≥ 100,000 cells/mm^3^, white blood cell ≥ 3000 cells/mm^3^, absolute neutrophil count ≥ 1500 cells/mm^3^), liver function (transaminases ≤ 3 times the upper limit of normal [ULN], bilirubin ≤ 1.5 times ULN), and renal function (creatinine ≤ 1.5 times the ULN and urine protein/creatinine ratio ≤ 1). Patients with uncontrolled hypertension defined as ≥145/90 were not eligible. This study was approved by the Northwestern University Institutional Review Board. All patients were required to provide signed informed consent before enrollment and were aware of being part of an investigational protocol.

### 2.2. Study Design

This open-label, Simon two-stage, phase 2 study was designed to evaluate the efficacy, safety, and tolerability of Bev in adult patients with brain metastases who have failed WBRT. Bev was administered at a dose of 10 mg/kg intravenously every 2 weeks, with one cycle of treatment defined as 28 days (or approximately 4 weeks). Treatment was continued until clinical or radiologic progression or severe toxicity.

Patients were evaluated for response through contrast-enhanced brain MRIs, computed tomography of the chest, abdomen, and pelvis (CT CAP), and clinical assessment at baseline and then every 8 weeks until disease progression. Brain imaging response was evaluated based on standard Response Assessment in Neuro-Oncology (RANO) criteria. Physical and neurologic exam results, along with KPS performance status, were assessed on the first day of each cycle. The Functional Assessment of Cancer Therapy-Brain (FACT-BR) is a validated tool to assess the quality of life in patients with brain metastases [14]. Patient-reported outcomes were assessed with FACT-Br at baseline and then at the start of every other cycle on the same day after surveillance imaging.

The primary endpoint was to assess objective radiologic tumor response using RANO. Secondary endpoints were assessing progression-free survival at 6 months (PFS6), time to progression, time to response, duration of response, overall survival (OS), and safety. Adverse events were assessed using the Common Terminology Criteria for Adverse Events (CTCAE) version 4.03.

### 2.3. Statistical Considerations

The primary outcome, radiographic response rate, was estimated using the Atkinson and Brown confidence limits [15]. To estimate time to progression, progression-free survival, and overall survival, Kaplan–Meier curves were calculated. PFS6 was determined from the progression-free survival curve. Time to response in responders was also estimated using a Kaplan–Meier curve. Duration of response in responders was estimated as the time from response to progression, and has been summarized using descriptive statistics such as median and range. Safety data have been summarized using frequencies and percentages, and adverse events were characterized by type, frequency, timing of occurrence, and attribution. While on treatment, changes in FACT-Br were analyzed using a longitudinal linear model that estimated the population averaged mean score as a function of time.

A Simon two-stage optimal phase 2 clinical trial design was used. Sample size calculations yielded a total of 24 patients, with 9 used for the first stage. If 1 or more responses were observed in the first 9 patients, an additional 15 patients were to be enrolled to reach a total of 24 evaluable patients. If 3 or more responses were observed, it would be evident that the regimen was active with an overall response rate of 25%, with 90% probability (power = 0.90) at the 1-sided 0.10 alpha level. To account for potential dropouts or ineligible patients, a total of 27 patients were planned for enrollment to ensure at least 24 evaluable patients.

## 3. Results

### 3.1. Patient Characteristics

Twenty-seven (27) patients were enrolled in this study. Twenty-four were women and three were men, with a median age of 54 (range 27–73). The median KPS was 80 (range from 70 to 100) at enrollment. Twenty-one patients had breast cancer (78%), four had non-small-cell lung cancer (15%), one had neuroendocrine cancer (3%), and one had papillary serous adenocarcinoma of the fallopian tube (3%). The median time from completion of WBRT to study enrollment was 290 days (range from 49 to 1285). The median number of prior treatment regimens prior to enrollment were 6 (range from 3 to 15). The median number of treatment cycles in this study were 5.5 (range from 1 to 20). The median follow-up time was 8.7 months (range from 2.4 to 47.9). One patient was lost to follow-up. 

### 3.2. Radiologic Response

**Figure 1:**
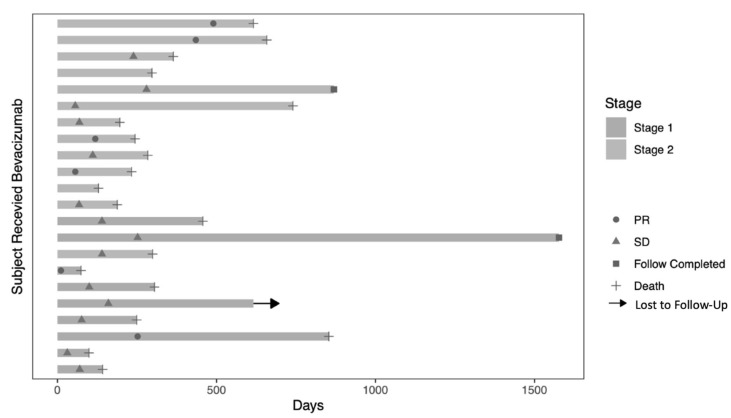
Swimmer plot of duration of response as measured from first treatment to date of study.

Of the 27 treated patients, 24 patients had evaluable data for response assessment. The best radiologic response per the RANO criteria was partial response (PR) in 2 patients (8.33%), though 16 patients (66.7%) had stable disease (SD). Clinical benefit (PR + SD) was seen in 18 patients (75%). The median duration of response for the 18 patients with clinical response was 6.7 months. Patients with PR demonstrated a median duration of response of 15.2 months and 6.4 months for those with SD. (Figure 1, swimmer plot of duration of response).

### 3.3. Survival

**Figure 2:**
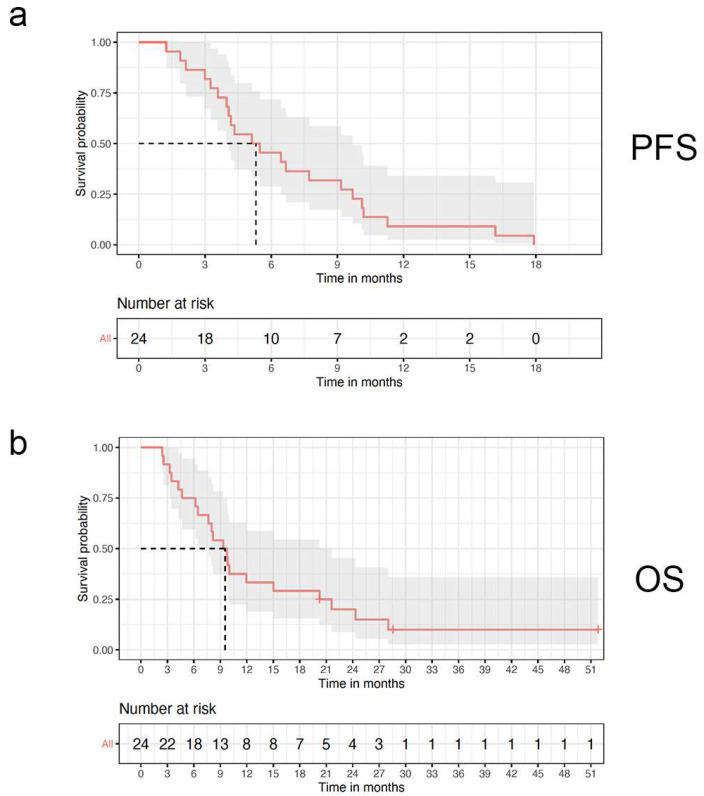
Kaplan–Meier curves for (**a**) progression-free survival (PFS); (**b**) overall survival (OS). Dashed lines indicate median survival.

PFS6 was 46%. The median PFS was 5.3 months (range from 3.3 to 7.7 months) and the median OS was 9.5 months (range from 6.3 to 15.0 months). Twenty-one patients died from systemic disease before documented neurologic progression. Ten patients had systemic progression while on therapy. The median time to progression based on imaging, neurologic progression, and increasing steroid requirements was 4.7 months (range from 2.1–16.2 months). (Figure 2, PFS and OS Kaplan–Meier curves).

### 3.4. Safety

Treatment was well tolerated, with toxicity rates as expected with standard of care Bev. There were minimal attributable grade 3 toxicities including hypertension (n = 6), lymphopenia (n = 6), headache (n = 3), and thromboembolism (n = 1). No dose reductions were made.

### 3.5. Quality of Life

**Figure 3:**
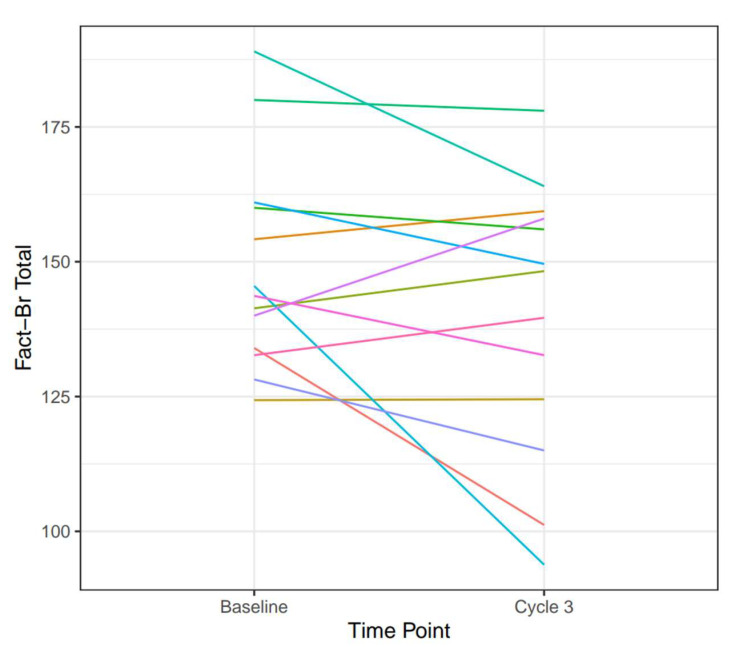
Directionality chart of quality of life with Functional Assessment of Cancer Therapy-Brain (FACT-Br) total score.

Thirteen patients (48%) completed FACT-Br at baseline and at cycle 3. For these patients who completed sequential quality of life (QOL) assessments, there was no significant decline in QOL, with a mean FACT-Br Cancer Score, General Score, Trial Outcome Index, and Total score of 65.7, 83.0, 106, and 148.8 at baseline and 63.6, 76.4, 99.4, and 140.0 at cycle 3, respectively. There was no clear pattern correlating FACT-Br scores with the extent or time of disease response (Figure 3, directionality chart for FACT-Br total score).

## 4. Discussion

For patients who are not candidates for stereotactic radiosurgery, WBRT is an important tool in brain metastasis management to achieve intracranial disease control and to palliate neurologic symptoms. After WBRT, patients often experience intracranial progression after several months [16,17]. There are limited data on effective treatment options for patients whose brain metastases progress after WBRT, posing a significant clinical challenge. For select patients, treatment options include stereotactic radiosurgery, surgery for symptomatic lesions, and rarely, a second course of WBRT [5,18,19,20]. There are limited data on systemic therapies in this population; however, patients may benefit from molecularly targeted therapies and novel peptide–drug conjugates [21,22,23]. It is possible that patients may respond to molecular targeted therapies with brain penetrance and immunotherapy; however, most systemic cancer agents lack the ability to cross the blood–brain barrier, and there is sparse research on the use of these drugs in this specific population. In particular, at the time of this study’s development and early enrollment, these molecularly targeted drugs were not widely available in clinical practice. Furthermore, these agents are only available to a small subset of patients with targetable markers, and thus, a more generalized treatment option that does not require a specific molecular target is needed.

The best objectively confirmed radiologic response in this study was partial response in only 8.33% of patients; thus, this study did not meet its primary endpoint. Although not meeting the primary endpoint, treatment with Bev resulted in a meaningful clinical benefit defined as achieving stable disease or better. In this heavily pretreated patient population with multiple primary histologies, the median PFS was 5.3 months and the median OS was 9.5 months, which is an improvement compared to historical controls of a median survival of 3–6 months after WBRT [24]. Sixty-six percent of patients demonstrated the best radiologic response of stable disease. However, it is important to note that this response was durable at approximately 6.6 months, demonstrating no radiologic evidence of new metastases. Patients were able to continue a variety of systemic therapies (Table 1) while receiving Bev, which likely contributed to this improvement in survival, as only three patients had neurologic progression before death, demonstrating the importance of controlling neurologic symptoms in addition to systemic disease burden. Of note, there was no notable added toxicity when combining Bev with different systemic therapies. Treatment was very well tolerated with minimal attributable grade 3 toxicities.

This study with a subset of our patients being treated concurrently with Bev and other systemic agents targeting non-CNS diseases mirrors the “true practice setting” where patients need their brain metastases addressed simultaneously with their systemic disease. In this study, not only did Bev treatment show outcomes superior to historic controls, but most importantly, it was shown to be safe for our patients. This did not increase adverse events in our patients as compared to the known Bev profile. Additionally, and perhaps most importantly, patients did not experience a decline in their quality of life when Bev was added to their systemic regimen (please see Table 1 for specific concurrent therapies).

This durable radiologic response and improved survival may be due to the anti-angiogenic effect of Bev, which is supported by multiple studies in lung adenocarcinoma and breast cancer that have demonstrated decreased brain metastasis formation with Bev treatment [25,26,27,28,29]. This could be due to decreased seeding of the central nervous system and/or the prevention of growth of micrometastatic metastases into radiologically visible macrometastases. Additionally, it is difficult to ascertain if the radiologic response reflects true disease control or if it is primarily a pseudo-response phenomenon, as seen commonly in patients with high-grade gliomas, due to blood–brain barrier stabilization causing decreased contrast uptake, making small metastases not readily visible on imaging [30]. Aside from response rate, the OS of the patients in this study did show an improvement as compared to historic controls. In the 75% of the patients in this study who experienced clinical benefits, this was sustained for a median of 6.7 months.

The impact on QOL related to our treatments must be considered, especially in patients with heavily pretreated brain metastases, as symptom palliation is paramount in the case of incurable disease. We collected patient-reported measures by using the FACT-Br tool, which supported that Bev did not have any negative impact on QOL. A shortcoming of this study was limited robust and prolonged QOL metrics in all patients.

Most patients had stable to slightly lower FACT-Br scores, suggesting no statistically meaningful impact on QOL metrics. Furthermore, there was no clear association between length of stable radiologic disease and FACT-Br, which may be due to the heterogeneity of patients, histologies, systemic therapies, and time from prior WBRT. Future studies should ensure adequate assessment of this important outcome metric.

A key limitation of this study is the selection of radiologic response as the primary endpoint, reflecting our understanding of Bev and its clinical benefits at the time the study was developed. There is now a significant body of evidence and collective understanding within the field of neuro-oncology on the radiologic pseudo-response achieved with Bev and its impacts on outcomes in patients with high-grade gliomas [31]. In a contemporary setting, a radiologic endpoint would be secondary, with primary endpoints being survival, neurologic outcome, quality of life, and extent of edema control. Although this study allowed for multiple histologies, another limitation is the over-representation of breast cancer patients, at nearly 80%, in this cohort. Due to the small sample size of the other disease types, it is difficult to state if our results can easily be extrapolated to patients with other types of brain metastases, and thus, these would need to be assessed in a larger study.

## 5. Conclusions

Patients with brain metastases who progress after whole-brain radiotherapy have limited treatment options and poor outcomes. This is the first prospective study assessing the efficacy and safety of bevacizumab in patients with solid tumor brain metastases in this population. Our data demonstrated sustained radiologic response, decreased brain metastasis formation, and no negative impact on quality of life when compared to historical controls. Treatment was well tolerated, with minimal toxicities, even when combined with a variety of systemic therapies for different tumor types. Our study suggests that bevacizumab should be considered a treatment for patients with tumor progression after whole-brain radiotherapy as it is a safe and well-tolerated therapy that maintains quality of life in this heavily pretreated population who have limited treatment options.

## Figures and Tables

**Table 1:** Demographics and clinical characteristics (total patients n = 27).

Age (Years)	
Mean (SD)	52.5 (13.5)
Median (range)	54 (27, 73)
**Sex, no. (%)**	
Female	24 (88.9%)
Male	3 (11.1%)
**Race, no. (%)**	
American Indian or Alaska Native	1 (3.7%)
Asian	1 (3.7%)
Black	3 (11.1%)
White	22 (81.5%)
**Ethnicity, no. (%)**	
Hispanic	3 (14.8%)
Non-Hispanic	23 (85.2%)
**KPS**	
Mean (SD)	83.81 (8.05)
Median (range)	80 (70, 100)
**Primary Malignancy, no. (%)**	
Breast	21 (77.8%)
Estrogen Receptor (ER)+	9 (42.9%)
Human Epidermal Growth Factor Receptor (HER2)+	14 (66.7%)
Triple Negative (TNBC)	4 (19.1%)
Non-Small-Cell Lung Cancer (NSCLC)	3 (11.1%)
Epidermal Growth Factor Receptor (EGFR)	2 (66.7%)
Small-Cell Lung Cancer (SCLC)	1 (3.7%)
Neuroendocrine	1 (3.7%)
Papillary Serous Adenocarcinoma	1 (3.7%)
**Concurrent Systemic Treatment**	
No. (%)	14 (51.6%)
Trastuzumab	7 (26%)
Capecitabine	5 (19%)
Lapatinib	3 (11%)
Abraxane	3 (11%)
Eribulin	1 (3%)
Halaven	1 (3)%)
Kadcyla	1 (3%)
Osimertinib	1 (3%)
Paclitaxel	1 (3%)
Epratuzumab	1 (3%)

## Data Availability

The data presented in this study are available on request from the corresponding author.
